# Residual strength of granitic rocks: interplay between GSI and confining pressure

**DOI:** 10.1038/s41598-025-14419-9

**Published:** 2025-08-07

**Authors:** Samad Narimani, Seyed Mortaza Davarpanah, Ákos Török, Balázs Vásárhelyi

**Affiliations:** 1 Department of Engineering Geology and Geotechnics, Faculty of Civil Engineering, Budapest University of Technology and Economics, Budapest, Hungary; 2 Research Institute of Mines and Environment (RIME), Université du Québec en Abitibi-Témiscamingue (UQAT), Rouyn-Noranda, QC J9X 5E4 Canada

**Keywords:** Residual strength, Granite, Residual strength models, Triaxial testing, Engineering, Civil engineering

## Abstract

Residual strength is an important factor in rock mechanics and geotechnical engineering, referring to the remaining strength of rocks following considerable deformation or damage. This study investigates the post-failure behavior of granitic rock samples from Bátaapáti, Hungary, using multiple failure triaxial testing and residual strength models. Experimental results show that residual strength is significantly influenced by confining pressure, with values increasing as confinement rises. Key models, including the Hoek-Brown failure criterion, Geological Strength Index (*GSI*), Residual Strength Index (*RSI*), and Cohesion-Loss approaches, were evaluated for their ability to predict residual strength. Nonlinear regression of deviator stress versus confining pressure was found. Using this nonlinear model, the relationship between the residual GSI and the confining pressure could be determined. It was carried out that the increasing the confining pressure the residual GSI logarithmically increased. It means, the residual *GSI* is not a material constant.

## Introduction

Residual strength is a critical concept in rock mechanics and geotechnical engineering, representing the reduced strength of rock material after significant deformation or damage. Unlike peak strength, which refers to the maximum stress a rock can withstand before failure, residual strength pertains to the stress level the rock can sustain after substantial strain or when subjected to prolonged shear deformation. This parameter plays a crucial role in understanding the long-term stability and behavior of geological formations, particularly in contexts such as mining, tunneling, slope stability, and earthquake mechanics.

Modeling the post-failure behavior of rock masses requires an understanding of residual strength, particularly when the rocks are subjected to progressive failure mechanisms, high pressure, or cyclic stress. Several factors influence it, including the mineral composition of the rock, its original strength characteristics, degree of weathering, and the presence of faults or fractures. The examination of joint surfaces, gouge material, and fracture mechanics is essential because the residual strength in fractured or faulted rocks frequently corresponds with the shear strength of the rock’s discontinuities rather than the intact material. Cai et al.^1^ conducted simulations to analyze the yielding zones around a 6-meter-wide tunnel under varying residual strength parameters. Their findings highlighted the critical impact of residual strength on the extent and distribution of yielding zones. Consequently, accurately determining the residual strength of rock materials is essential for optimizing excavation design and ensuring stability during construction.

Researchers have identified three key methods for analyzing the post-peak mechanical behavior of rocks, revealing that under specific confining pressure conditions, rocks demonstrate strain softening and retain a degree of residual stress during the post-peak phase. This highlights the complex nature of rock deformation beyond the peak strength^2^. Several other researchers have reviewed methods to determine the residual strength of rocks, emphasizing triaxial compression tests performed under different confining pressures. The resulting residual strength values are then analyzed and fitted using models such as Mohr-Coulomb (M-C) or Hoek-Brown (H-B) to derive key strength parameters. This approach provides a systematic way to characterize rock behavior in post-failure conditions. Various studies have concluded that residual strength is typically low, often approaching zero in unconfined tests, while significantly increasing under higher confining pressures. This demonstrates the strong influence of confinement on the post-failure strength of rocks^3,4^. Gao and Kangas^5^ demonstrated that as confining pressure increases, the resulting rise in residual strength is more pronounced than the corresponding increase in peak strength. This important finding aligns with empirical field observations conducted in underground coal pillars, where it was noted that the ratio of residual strength to peak strength grows progressively with increasing depth into the pillar.

Mineralogical composition, weathering, fracture networks, confining pressure, and loading rate are some of the variables that affect a rock’s residual strength. Additionally, certain laboratory and field methods are required to measure the residual strength of rocks. Ring shear tests, triaxial tests, and direct shear tests are examples of common techniques.

Residual strength has long been recognized as measurable through triaxial testing, with numerous studies indicating that it is an inherent material property influenced by the type of rock^6–8^. This understanding highlights the connection between residual strength and rock characteristics, emphasizing the critical role of rock type in defining post-failure behavior.

Over the past two decades, numerous researchers have explored the concept of residual strength in rock masses and developed various models to estimate and predict it^1,9–20^. These studies have employed a variety of approaches, including in situ testing, numerical modeling, and analytical methods, to gain insights into the post-failure behavior of rock masses. The models proposed aim to refine the understanding of how rock masses retain strength after reaching peak conditions, often leveraging established frameworks such as the Hoek–Brown failure criterion or Geological Strength Index (*GSI*) modifications. Despite these advancements, the practical implementation and widespread acceptance of these models within engineering design and construction projects have been slow. This lag is often attributed to the complexity of the models, the variability of geological conditions, and the challenges associated with accurately gathering in situ data. Nonetheless, these contributions form a crucial foundation for advancing rock mechanics and improving the long-term stability assessment of rock masses.

Numerous models have been developed to estimate the peak and residual strengths of intact rock, reflecting advancements in rock mechanics. Key models include the Hoek-Brown (H-B) model^21^, the Mohr-Coulomb (M-C) model^22^, and the Joseph-Barron (J-B) model^23^. Other significant contributions include the *GSI*-based model^1^, the GSI-softening model^24^, the cohesion loss model^3^, and the GSI-constant confining pressure model^17,18^. These models provide critical frameworks for understanding and predicting rock strength under various geological conditions.

Mahmutoglu and Şans^25^ showed that the strength of brittle rock at shallow depths can be effectively estimated using a simplified quadratic equation. They found that the residual strength parameters of the tested material vary significantly with the range of confining pressure applied. Additionally, triaxial tests on previously cracked marble revealed that increasing confining pressure caused a sharp decrease in the internal friction angle, while residual cohesion increased considerably under high confining pressures. These results highlight the influence of confining pressure on the mechanical behavior of brittle rocks, particularly in high-pressure environments. The incorporation of stress-sensitive elastic parameters offers a more complete framework for modeling the mechanical behavior of rocks under both peak and residual conditions^26^. Cai et al.^1^ introduced a method to approximate the residual strength of rock masses by formulating a residual *GSI*. This method was derived from in situ block shear tests and back analyses of rock slope stability. Their findings revealed that the residual strength of hard, jointed rock masses aligned closely with the residual strength of intact rocks, as determined through triaxial testing. Finally, they proposed a formula to calculate the residual Geological Strength Index (*GSI*_*r*_) based on the initial *GSI* value, where a fully intact rock mass corresponds to a *GSI* of 100. This residual *GSI* can then be applied to estimate the rock mass’s residual strength using the generalized Hoek–Brown criterion^27^. This approach provides a practical means of evaluating the long-term stability and strength of rock masses following deformation or failure.

He et al.^2^ introduced a new approach for predicting rock residual strength using the Hoek-Brown (H-B) failure criterion. The model relies on a single parameter to regulate the nonlinearity of residual strength. Predictions for limestone, granite, slate, and sandstone closely match laboratory test results, demonstrating the method’s accuracy. Moreover, He et al.^28^ proposed two criteria using linear elastic fracture mechanics to predict the residual strength and brittle-ductile transition (BDT) point in brittle rock. The study examines how microcrack and slip friction affect these properties, showing the criteria’s effectiveness in providing insights into brittle-ductile transition mechanics. Also, in evaluating the residual strength of brittle rocks, the transition stress defined through the Hoek–Brown failure criterion and Mogi’s brittle-ductile transition limit, as proposed by Davarpanah et al.^29^, provides essential insight into the mechanical behavior of rocks under varying confining pressures.

Confining pressure plays a significant role in determining the residual strength of rock masses. As confining pressure increases, the residual strength also increases, transitioning from brittle failure to ductile behavior. This relationship is particularly evident in granitic rocks, where higher confining pressures lead to a more pronounced increase in residual strength compared to peak strength^30^. The transition from brittle to ductile behavior is characterized by a shift from crack-dominated failure to frictional sliding and plastic flow, which is influenced by the confining pressure and the *GSI* of the rock mass.

**Fig. 1:**
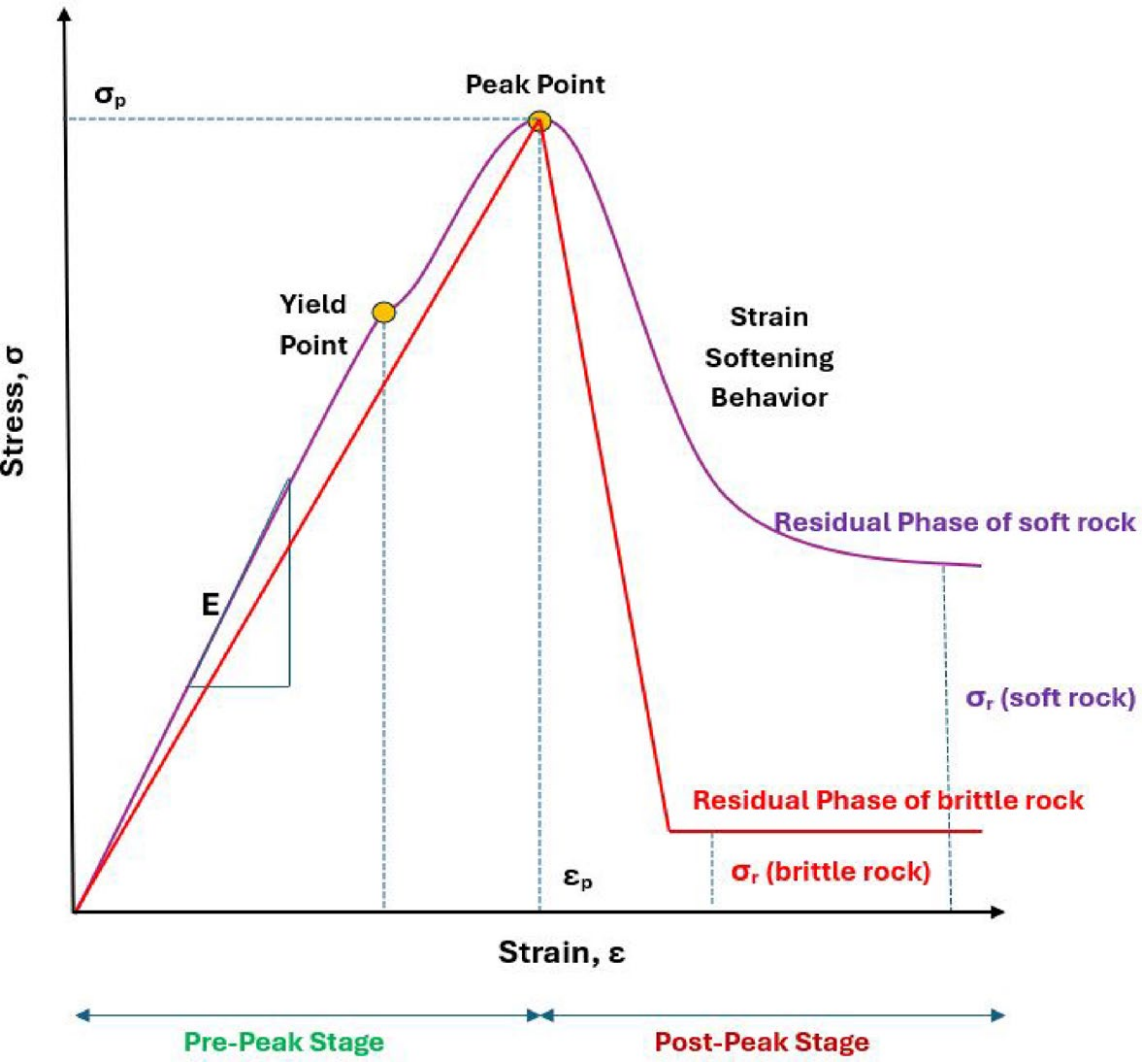
Stress-strain behavior of brittle and soft rocks, illustrating pre-peak (elastic and peak strength), post-peak (strain-softening), and residual strength stages, showing σp (peak/failure strength), εp (peak strain), and σr (residual strength).

Research conducted by numerous scholars^31,32^ suggests that the stress-strain behavior of rocks can be broadly categorized into four stages, as depicted in Fig. 1:

1. Compression Phase: During the early stage of loading, the cracks within the rock slowly close. In this phase, the tangent modulus of the rock progressively increases until it stabilizes upon reaching the elastic stage.
2. Elastic Stage: This stage begins once the majority of the cracks in the rock have closed. During this phase, the tangent modulus remains relatively constant. However, as the rock transitions into the plastic stage, the tangent modulus starts to decline.
3. Plastic Stage: In this phase, new micro-cracks progressively develop within the rock. The tangent modulus steadily decreases during this stage, eventually approaching zero at the peak stress point.
4. Post-Peak Stage: Once the rock reaches its peak strength, its internal structure undergoes a sudden transformation. This stage concludes with either the residual stress phase or the complete failure of the rock.

The post-peak stage, particularly in brittle rocks, can be further interpreted as consisting of an initial rapid stress drop immediately after peak strength, followed by a more gradual decline toward the residual strength. This residual strength may be comparable to the constant volume strength described in soil mechanics under drained conditions.

The following three requirements should be met by a model used to estimate the strength of the rock residual^2^:

1. The model parameters are easily obtainable, have a straightforward shape, and have distinct physical meanings.
2. There is a substantial nonlinearity in the rock’s residual strength envelope overall.
3. The origin of the coordinates in the principal stress space should be traversed by the residual strength envelope, particularly for the majority of brittle rocks.

This study primarily emphasizes the intricate relationship between confining pressure and the Geological Strength Index (*GSI*) in the assessment and determination of a rock’s residual strength. The research underscores that residual strength is not an isolated parameter but rather one that is significantly influenced by variations in confining pressure, which in turn is closely linked to changes in the *GSI*. By adjusting the *GSI*, corresponding shifts in confining pressure can occur, ultimately affecting the magnitude of the rock’s residual strength. This highlights the nonlinear-value interplay between geological characteristics and mechanical constraints, reinforcing the importance of considering both factors when evaluating the post-peak behavior and long-term stability of rock materials.

## Materials and methods

### Triaxial test procedure

**Fig. 2:**
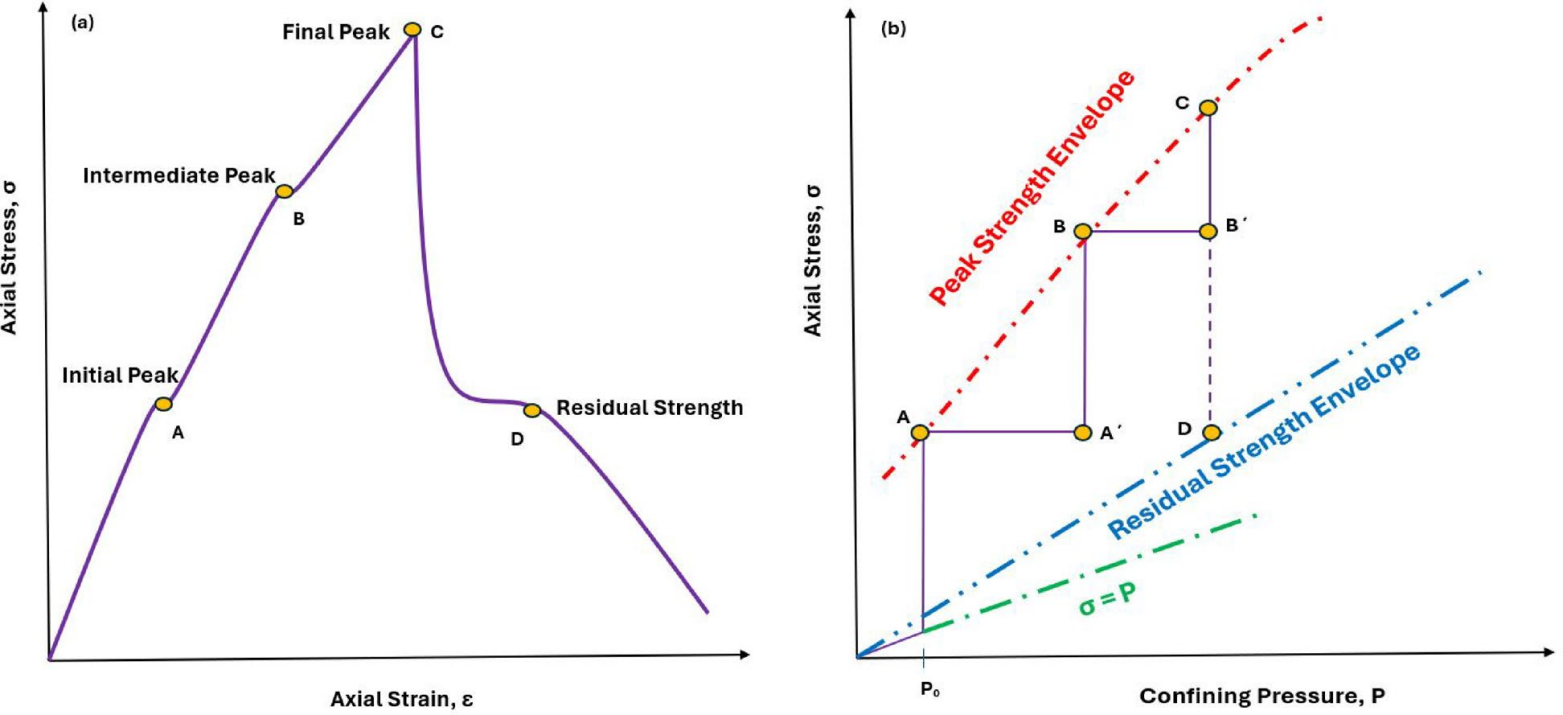
Multiple failure state triaxial test (a): The axial stress-axial strain curve; (b) the axial stress-confining pressure curve (Modified after ISRM, 1983).

The multiple failure state and strain controlled triaxial tests were developed by Kovári and Tisa^8^ and Kovári et al.^33^. This method is suggested for determining both the failure and residual strength of the intact rock^34^. The procedure is presented in Fig. 2: applying initial confining pressure (p_0_) the axial load is increased until the corresponding peak strength is observed in the axial stress-axial strain curve (Point A, Fig. 2a). The confining pressure is increased in one step (from A to A’ – Fig. 2b). This confining pressure is constant until the next peak strength is observed (Point B, Fig. 2a) – and so on. This stepwise procedure is continued until chosen point (Point C, Fig. 2) is reached. The confining pressure will then be kept constant while the axial loading is continued. This will cause failure and the axial stress will drop its residual value (Point D, Fig. 2). The confining pressure is continuously reduced until the specimen is completely unloaded – the axial stress versus confining pressure curve will follow the residual strength envelope.

**Fig. 3:**
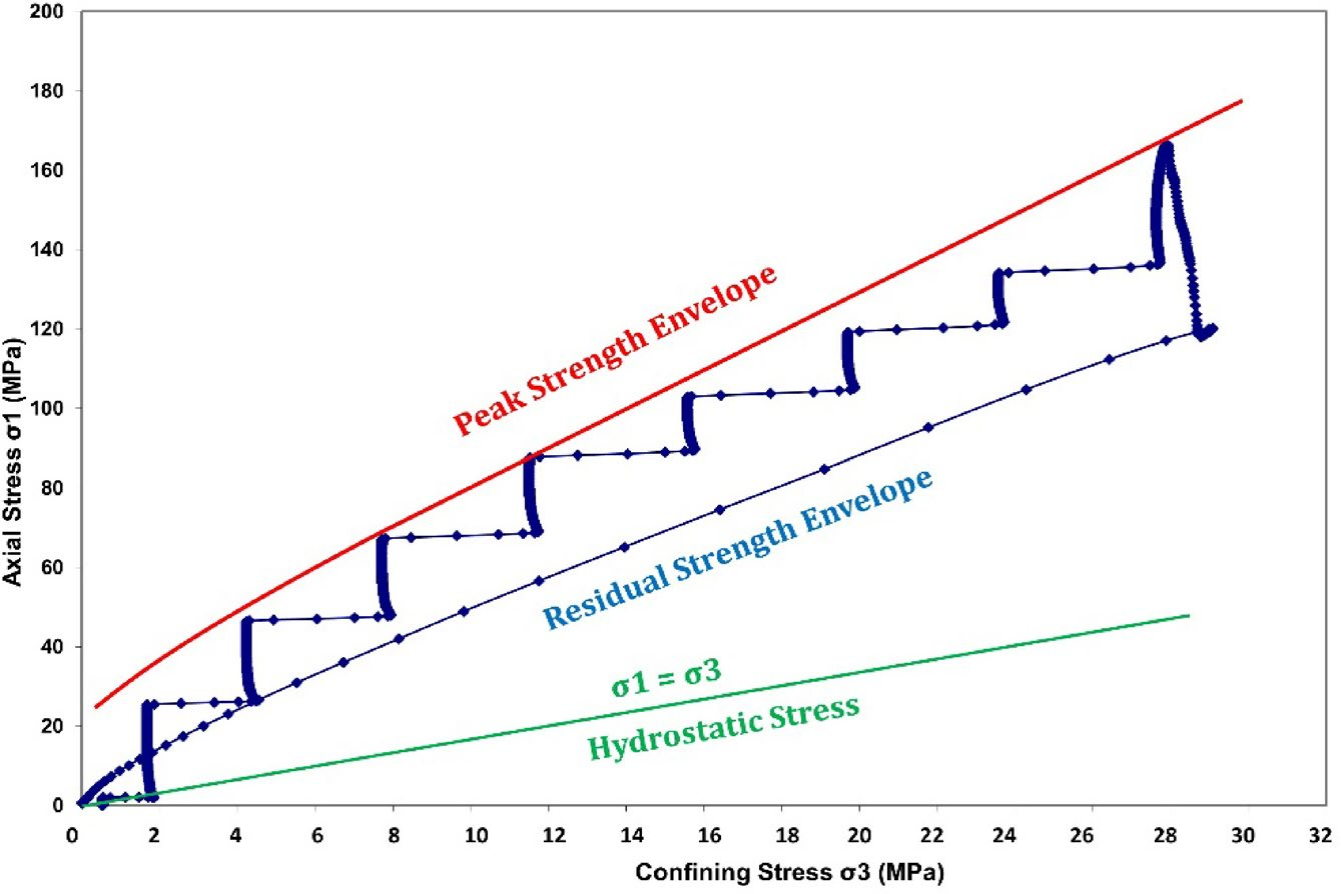
Measured axial stress – Confining pressure curve, using Multiple Failure State triaxial test.

Using the measurement peak results, the Hoek-Brown material constant (m_i_) can be determined for the intact rock sample^35^. An average measured axial stress-confining pressure curve is presented in Fig. 3. The figure shows the results of a multiple failure state (MFS) triaxial test. This type of test measures the strength of rock under increasing confining pressures, allowing for the evaluation of both peak and residual strengths in multiple stages. The stepwise increase in axial stress corresponds to successive stages of loading and failure at increasing confining pressures. The upper curve represents the peak strength values at each confining pressure stage, outlining the failure envelope of the rock, while the lower curve traces the post-failure or residual strength of the rock after initial peak failure, showing a progressively increasing trend but at a reduced slope. Moreover, the thin diagonal line near the bottom represents the condition where axial stress equals confining pressure, which acts as a reference for hydrostatic loading conditions (where no shear stress is present).

### Residual strength fitting approaches

According to Alejano et al.^36^, there are three potential approaches for fitting the residual strength based on peak strength for comparative purposes: the Hoek-Brown approaches (so called GSI and RSI approaches) and the cohesion-loss approach.

#### The GSI approach^1^

Cai et al.^1^ recognized that while the original Geological Strength Index (*GSI*) system effectively estimated peak strength, it lacked guidelines for assessing residual strength. To address this, they proposed a method that modifies the GSI values to account for the conditions of rock masses at residual states. Their approach involves adjusting the GSI value based on the degree of disturbance and the characteristics of the rock mass post-failure.

Similar to the original *GSI* system, the extended approach evaluates the structure of the rock mass and the condition of discontinuity surfaces. However, for residual strength estimation, it emphasizes the altered conditions after significant deformation. Therefore, they introduced a method to adjust the *GSI* values to reflect the reduced interlocking and increased surface degradation associated with residual states. This adjustment is crucial for accurately estimating the residual strength parameters. Then, the modified *GSI* values are then incorporated into the Hoek-Brown failure criterion, a widely used empirical strength criterion for rock masses, to estimate the residual strength parameters. This integration allows for a more comprehensive analysis of rock mass behavior post-failure.

Cai et al.^1^ proposed an adjustment to the GSI value to reflect the residual state of the rock mass. The residual *GSI*, denoted as *GSI*_*r*_, is calculated using the following equation:

1 $$\:{GSI}_{r}=\text{G}\text{S}\text{I}{\text{e}}^{-0.0134GSI}$$

This equation reduces the original *GSI* value to account for the degradation in rock mass quality due to deformation.

Geological Strength Index (*GSI*) approach directly uses the Hoek-Brown failure criterion^27^ where the parameters m_b_, a and s are computed based on Eqs. (3–5), so the results only depend on the GSI parameter, which can be fitted to available residual strength data:

2 $$\:{{\upsigma\:}}_{1}-{{\upsigma\:}}_{3}\:=\text{U}\text{C}\text{S}{\left(\frac{{m}_{b}}{UCS}{{\upsigma\:}}_{3}+s\right)}^{a}$$

Where: σ_1_ and σ_3_ are the major and minor effective principal stresses at failure, respectively. UCS is the uniaxial compressive strength of the intact rock in its residual state. m_b_, s, and a are the residual material constants, derived from the adjusted *GSI*_*r*_.

The residual material constants (m_b_, s, and a) are essential for applying the Hoek-Brown criterion to residual strength estimation. These constants are determined using the following relationships:

3 $$\:{\text{m}}_{b}\:={\text{m}}_{i}{e}^{\frac{GSI-100}{28}}$$

4 $$\:\text{s}\:={e}^{\frac{GSI-100}{9}}$$

5 $$\:\text{a}\:=\frac{1}{2}+\frac{1}{6}\left({e}^{\frac{-GSI}{15}}-{e}^{\frac{20}{3}}\right)$$

The goal of the original theory of Cai et al.^1^ was to determine the residual strength of the rock mass, but it can be also used for estimating the residual strength based on laboratory tests results, as well^17,18^.

#### The RSI approach^17,18^

The Residual Strength Index (*RSI*) approach, introduced by Walton et al. in 2019^17^ and further refined in 2021^18^, offers a methodical framework for evaluating the residual strength of rock masses. This approach adapts the widely recognized Geological Strength Index (*GSI*) system to better represent the post-peak behavior of rocks, particularly under conditions where they have undergone significant deformation or damage.

Walton et al.^18^ recognized the limitations of existing models in addressing the residual strength of rock masses. To address this, they proposed the *RSI* as an adaptation of the *GSI* system, specifically tailored for residual strength assessment. The *RSI* approach modifies the original Hoek-Brown criterion by setting the constant ‘s’ to zero, simplifying the equation and making it more applicable to conditions where the rock mass has undergone significant degradation.

In the RSI approach, the constant s is set to zero to reflect the loss of cohesion in the residual state. Additionally, the m_b_ parameter — which governs the frictional response in the Hoek–Brown criterion — is also reduced, as it is computed from the residual RSI value. This ensures that both cohesion and friction are appropriately degraded to reflect post-failure rock mass behavior.

By setting the constant ‘s’ to zero in the Hoek-Brown criterion, the *RSI* approach reduces the complexity of calculations, focusing on the most critical parameters that influence residual strength. This modification simplifies the Hoek-Brown equation and the related constants to:

6 $$\:{{\upsigma\:}}_{1}-{{\upsigma\:}}_{3}\:=\text{U}\text{C}\text{S}{\left(\frac{{m}_{b}}{UCS}{{\upsigma\:}}_{3}\right)}^{a}$$

7 $$\:{\text{m}}_{b}\:={\text{m}}_{i}{e}^{\frac{RSI-100}{28}}$$

8 $$\:\text{a}\:=\frac{1}{2}+\frac{1}{6}\left({e}^{\frac{-RSI}{15}}-{e}^{\frac{20}{3}}\right)$$

This adjustment allows for a more accurate estimation of the residual strength by focusing on the behavior of the rock mass after failure^17,18^.

In their method, the RSI maintains a conceptual similarity to the *GSI* system, allowing practitioners familiar with GSI to adopt the RSI approach with relative ease. This alignment facilitates a smoother transition and integration into existing rock mass assessment practices.

Studies have demonstrated that the RSI approach provides a good fit for residual strength data across various rock types, enhancing its credibility and applicability in diverse geological settings^17^.

The *RSI* value is expected to be equal to or greater than the *GSI* value^36^.

#### Cohesion-loss model^3^

Peng and Cai^3^ identified limitations in existing models when applied to residual strength determination. To address these, they proposed the Cohesion-Loss (C-L) Model, which introduces a parameter, denoted as λ, to represent the reduction in cohesion as deformation progresses. This parameter effectively captures the nonlinear behavior of the residual strength envelope, providing a more accurate representation of post-peak rock behavior.

The C-L model modifies the original Hoek-Brown criterion by incorporating the λ parameter, which adjusts the cohesion component to reflect its degradation post-failure. This adjustment allows the model to accurately represent the residual strength envelope of intact rocks.

In the C-L model, the parameter ‘s’ is modified to represent the residual strength state, denoted as ‘s_r_’. This modification reflects the loss of cohesion as the rock undergoes damage. The residual strength criterion becomes:

9 $$\:{{\upsigma\:}}_{1}-{{\upsigma\:}}_{3}\:={\left(\frac{{m}_{b}{{\upsigma\:}}_{3}}{UCS}+{s}_{r}\right)}^{a}$$

The parameter ‘s_r_’ is defined as:

10 $$\:{s}_{r}\:={{\uplambda\:}\:\times\:\left({{\upsigma\:}}_{3}/UCS\right)}^{a}$$

where ‘λ’ is a dimensionless parameter that characterizes the rate of cohesion loss with increasing confining pressure.

Therefore, Peng and Cai^3^ proposed the model -the cohesion loss (C-L) model- to describe the characteristics of the rock residual strength as below:

11 $$\:{{\upsigma\:}}_{1}-{{\upsigma\:}}_{3}\:={\left({m}_{b}UCS{{\upsigma\:}}_{3}\right)}^{0.5}$$

In this case they referred to the equivalent of residual m_b_ parameter as λ:

12 $$\:{{\upsigma\:}}_{1}-{{\upsigma\:}}_{3}\:={\left(\lambda\:{{\upsigma\:}}_{c}{{\upsigma\:}}_{3}\right)}^{0.5}$$

**Fig. 4:**
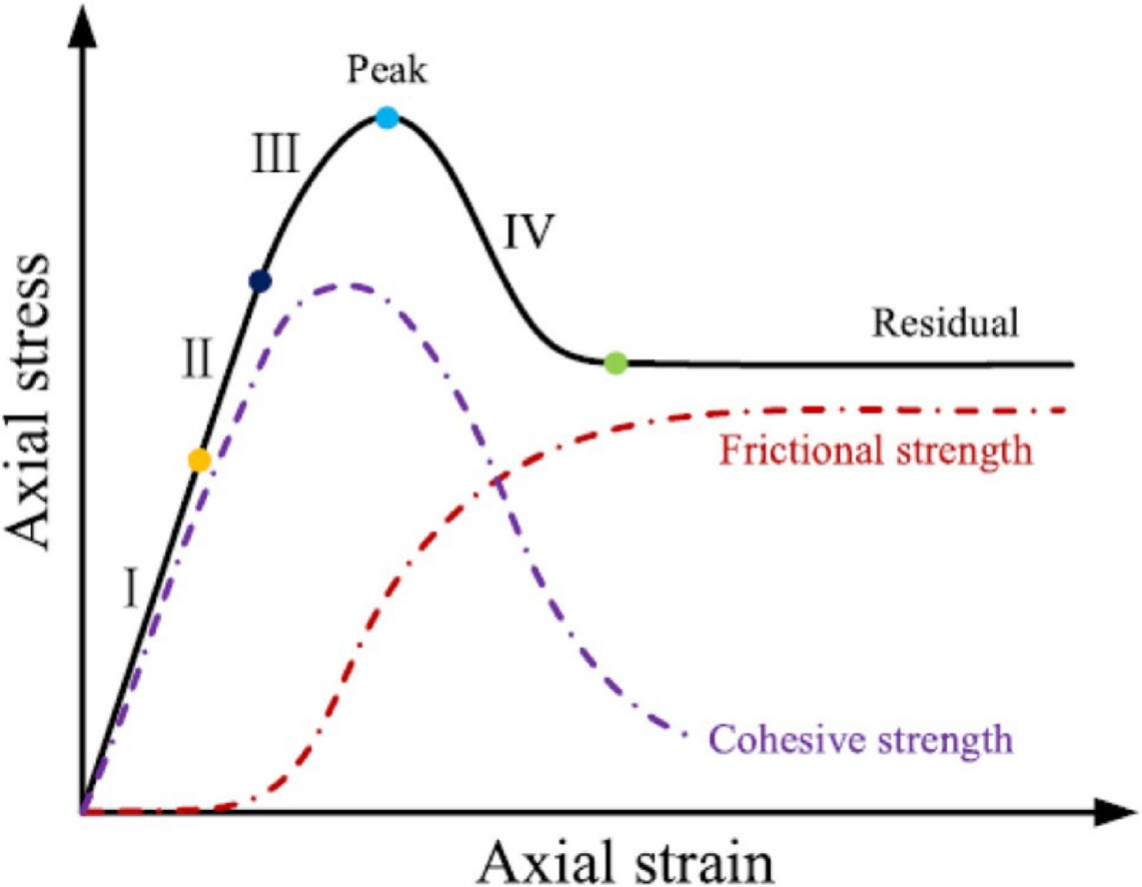
The mobilization of frictional strength and cohesive loss^3^.

The λ value can be obtained by representing residual strength date: plotting the (σ_1_ – σ_3_)^2^/UCS data in the function of the confining pressure (σ_3_) the slope of the fitting line through the origin is equal to λ. Figure 4 illustrates the deformation process of rock, which is known as cohesion loss and mobilization of frictional strength.

Peng and Cai^3^ validated their model using two sets of experimental data, demonstrating its capability to accurately represent residual strength envelopes across various rock types. Their analysis of 46 different rocks yielded an R² value greater than 0.9, indicating a strong correlation between the model’s predictions and observed data.

### Investigated rock

**Fig. 5:**
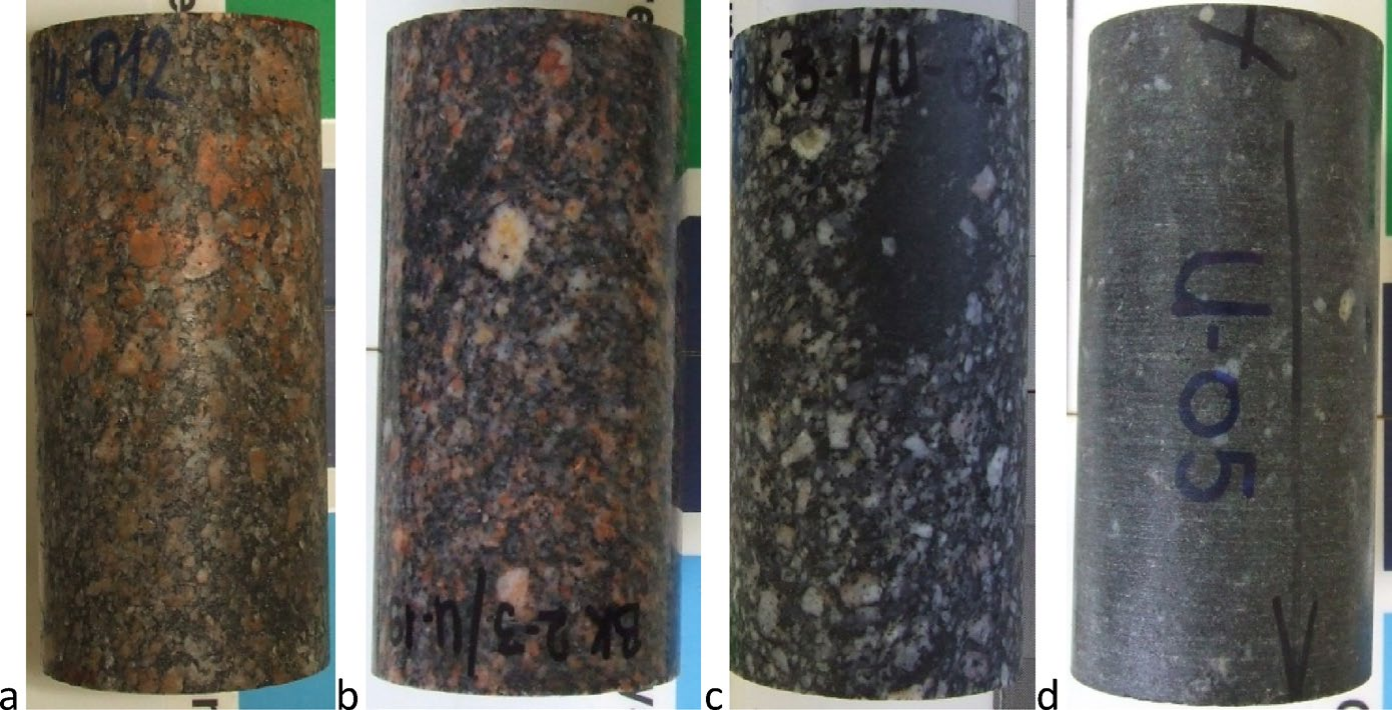
Main types of rock samples. a-b: megacryst-bearing, medium-grained, biotite-monzogranites, c:medium-grained, biotite-monzogranites with elongated monzonitic enclaves, d: quartz monzonite.

The investigated granitic rock samples originated from Bátaapáti (Hungary), the site of the low and intermediate radioactive waste repository currently under construction. This granite formation is a carboniferous intruded and displaced Variscan granite pluton situated in South-West Hungary. The main rock types are mainly microcline megacryst-bearing, medium-grained, biotite-monzogranites, and quartz monzonites (Fig. 5). The mechanical behaviors of the intact rock of this rock type were investigated and published by Davarpanah et al.^37,38^. These publications focused on the uniaxial compressive tests results. Parallel the huge number of uniaxial compressive tests, Multiple Failure State (MFS) triaxial tests were carried out for determining the failure envelope of this granitic rock. These results were initially analyzed by Vásárhelyi et al.^39^.

38 multiple failure triaxial tests were carried out for determining both the Hoek-Brown constant (m_i_) of the intact rock and the above introduced residual parameters (such as GSI, RSI and m_b_ values). The measured peak values (i.e. uniaxial compressive strength and mi parameter) and the residual strength constants according to the different approaches are summarized in Table 1 including the *GSI* approach^1^ (Eqs. 1–5), the RSI approach^18^ (Eqs. 6–8) and the λ approach^3^ (Eqs. 9–12). The *GSI* and *RSI* approaches provide closely aligned estimates of rock mass quality, with average values of 54 and 55, respectively, suggesting consistent results in predicting rock strength. The minimum *GSI* of 29 and maximum of 79, along with *RSI* values ranging from 26 to 81, reflect a wide range of rock mass conditions, from highly fractured to more intact formations. Standard deviations of 11 for GSI and 12 for RSI indicate moderate variability among the samples.

The λ approach, which quantifies deformation behavior and crack propagation, shows a broader spread, with an average value of 4.569, a minimum of 1.181, and a maximum of 15.069. This wide range and higher standard deviation (2.607) suggest significant differences in post-failure behavior across samples, potentially highlighting the heterogeneous nature of granitic rock. The relatively high maximum λ value indicates that some samples exhibit considerable resistance to deformation, while the low minimum reflects more brittle characteristics in others.

**Table 1:** Measured constants for both peak and residual constants of the investigated granitic rock.

No.	Peak Strength		Residual Strength		
	UCS	m_i_	GSI approach	RSI approach	λ approach
	(MPa)				
1	68.6	30.58	54	54	6.226
2	56.9	28.76	79	81	15.069
3	80.2	20.48	51	51	3.668
4	55.0	29.63	54	55	6.246
5	103.5	22.31	56	57	5.440
6	117.1	27.82	70	72	11.275
7	62.1	17.05	68	69	6.066
8	82.7	23.26	46	46	3.573
9	75.2	18.85	56	56	4.140
10	118.9	17.25	52	53	3.399
11	69.0	23.22	59	59	5.683
12	160.0	35.52	46	47	5.768
13	87.2	24.85	40	40	3.011
14	86.4	16.59	72	74	6.654
15	75.3	23.44	50	50	4.068
16	137.4	11.22	60	62	3.020
17	54.8	14.68	74	76	6.778
18	97.1	24.15	42	42	3.261
19	109.9	20.55	46	47	3.285
20	180.7	15.85	39	40	1.907
21	112.0	21.16	48	48	3.431
22	112.3	10.95	51	52	2.057
23	152.3	10.45	61	65	3.185
24	84.5	19.33	48	48	3.348
25	78.7	14.42	74	76	6.623
26	140.9	12.08	51	52	2.240
27	133.5	23.71	42	42	3.259
28	162.7	16.24	29	29	1.181
29	110.0	24.17	57	58	5.854
30	112.4	11.45	66	68	3.721
31	79.1	30.82	49	49	5.482
32	96.8	16.17	51	52	3.123
33	130.6	15.10	56	56	3.624
34	131.1	16.26	40	43	2.215
35	108.1	22.88	55	55	4.781
36	153.9	14.75	47	47	2.311
37	151.9	18.50	65	67	6.063
38	104.7	12.95	53	54	2.604
Av	106.15	19.93	54	55	4.569
STD	33.35	6.28	11	12	2.607
min	54.84	10.45	29	29	1.181
max	180.67	35.52	79	81	15.069

## Results

### Residual strength of the investigated intact rock

Initially, the residual strength of the intact rock was examined. The following non-linear regression was found plotting the deviator stress in the function of the confining pressure. Equation 13 was developed based on the residual strength data from 38 multiple failure state (MFS) triaxial tests. For each sample, the deviator stress (σ₁ − σ₃) was plotted as a function of the confining pressure (σ₃), and a nonlinear regression was fitted to derive the material constants b and c. The fitted constants for each test are presented in Table 2.

13 $$\sigma _{{\text{1}}} {-}\sigma _{{\text{3}}} = {\text{ b}}\sigma _{{\text{3}}} ^{{\text{c}}}$$

**Fig. 6:**
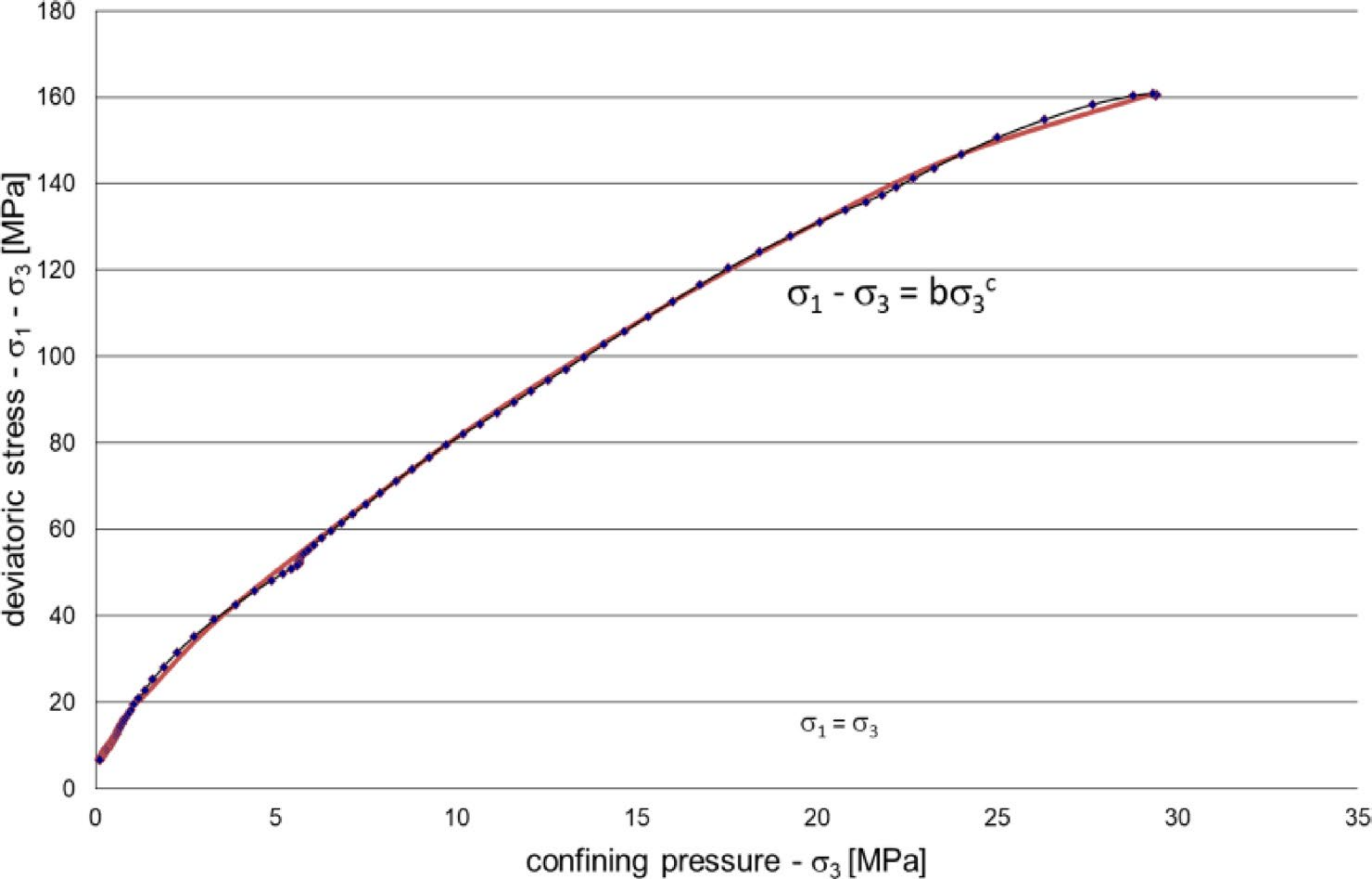
Deviatoric stress in the function of the confining pressure in case of measured residual strength results.

Where and are material constants that reflect the strength characteristics of the rock. Figure 6 visually illustrates the correlation between deviator stress and confining pressure, highlighting the trend of increasing stress with rising confinement. The derived equation captures the non-linear nature of the relationship, indicating that as confining pressure increases, the deviator stress grows at a diminishing rate, governed by the exponent . This reflects the rock’s transition from brittle failure to ductile behavior under higher confining pressures. – they are summarized in Table 2.

**Table 2:** Independent material constants related to the deviator stress in the function of the confining pressure.

No.	b	c
1	13.34	0.642
2	17.43	0.667
3	9.87	0.676
4	10.57	0.751
5	12.87	0.683
6	17.76	0.745
7	11.85	0.652
8	10.37	0.657
9	10.17	0.673
10	10.52	0.703
11	7.79	0.793
12	15.75	0.732
13	10.47	0.653
14	17.40	0.609
15	11.65	0.629
16	12.33	0.659
17	10.71	0.726
18	8.83	0.721
19	9.05	0.735
20	9.42	0.709
21	13.89	0.607
22	9.12	0.663
23	12.05	0.702
24	7.49	0.754
25	11.03	0.726
26	10.78	0.664
27	10.04	0.720
28	10.76	0.609
29	10.91	0.766
30	13.22	0.646
31	9.16	0.757
32	8.53	0.722
33	11.60	0.699
34	8.49	0.720
35	12.20	0.700
36	10.91	0.667
37	14.43	0.734
38	9.49	0.679
Av	11.38	0.693
STD	2.57	0.047
min	7.49	0.607
max	17.76	0.793

Constants *b* and *c* show an average value of 11.38 and 0.693, respectively. The standard deviation (STD) for *b* is 2.57, indicating moderate variability across the samples, while the STD for *c* is relatively low at 0.047, reflecting more consistent results in the exponent. The minimum values of *b* and *c* are 7.49 and 0.607, while the maximums are 17.76 and 0.793, respectively. This range highlights the diversity in rock behavior, with higher b values corresponding to samples capable of sustaining greater stress, while lower *c* values signify a more linear relationship between deviator stress and confining pressure. The distribution of the parameters b and c indicates that most samples show moderate residual strength, with a few outliers reflecting either notably strong or weak rock characteristics.

Using cohesion loss model, suggested by Peng and Cai^3^ which reflects how confining pressure influences the post-peak behavior of rocks, providing a more nonlinear-value interpretation of rock strength than conventional constant-value approaches:

14 $$\:\text{b}\:{{{\upsigma\:}}_{3}}^{\text{c}}\:={\left({\text{m}}_{\text{b}}\text{U}\text{C}\text{S}{{\upsigma\:}}_{3}\right)}^{0.5}$$

Equation (15) to (18) expand on this concept by introducing a logarithmic relationship between *GSI* and confining pressure. Equation (15) expresses *GSI* as a function of constants x and z, while Eqs. (16) and (17) define these constants based on material properties and stress conditions. Specifically, x links the *GSI* evolution to the exponent c from the deviator stress equation, while z incorporates key strength parameters such as b (a material constant), m_i_ (Hoek-Brown constant of intact rock), and uniaxial compressive strength (UCS). Equation (18) introduces adjustments for the Hoek-Brown parameter m_b_, suggesting that both *GSI* and m_b_ vary under different confinement levels, challenging earlier assumptions that GSI remains constant. Moreover, we used the Hoek et al.^27^ version of the GSI system, with modifications proposed by Cai et al.^1^ and further adjusted based on our empirical data.

15 $${\text{GSI }} = {\text{ x ln }}(\sigma _{3} ){\text{ }} + {\text{ z}}$$

16 $${\text{x }} = {\text{ 28}}\left( {{\text{2c }}{-}{\text{ 1}}} \right)$$

17 $${\text{z }} = {\text{ 1}}00{\text{ }} + {\text{ 28}}*\left( {{\text{2ln}}\left( {\text{b}} \right){\text{ }}{-}{\text{ ln}}\left( {{\text{m}}_{{\text{i}}} } \right){\text{ }}{-}{\text{ ln}}\left( {{\text{UCS}}} \right)} \right)$$

18 $$\:\:{m}_{b}=\frac{{b}^{2}}{UCS}\:{{{\upsigma\:}}_{3}}^{2c-1}$$

The formulation of Eqs. (15)–(18) is based on combining the nonlinear regression form of the deviator stress–confining pressure relationship (Eq. 13) with the Hoek–Brown failure criterion. In this approach, constants x and z were empirically defined to reflect how confining pressure influences GSI. Specifically, x is related to the curvature of the residual strength envelope (via the exponent c), while z incorporates material properties including b, m_i_, and UCS. This allows the GSI to vary logarithmically with σ₃, providing a confinement-sensitive estimation of residual strength. The approach preserves the structure of the Hoek–Brown formulation but introduces a nonlinear GSI suitable for post-failure conditions.

**Fig. 7:**
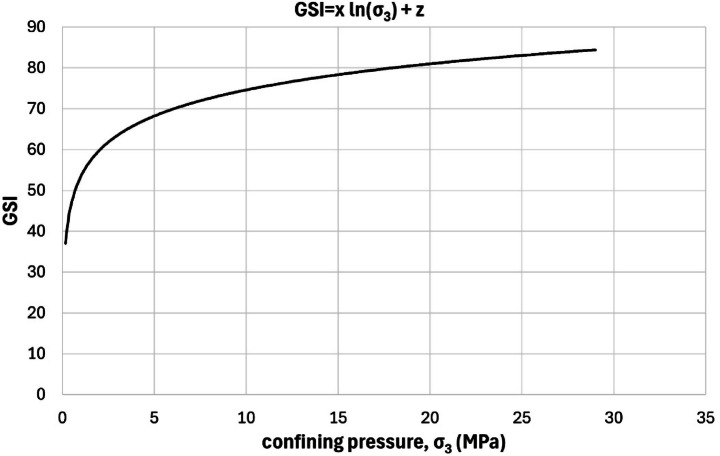
Relationship between *GSI*_r_ and m_b_ in function of confining pressure (σ_3_).

**Fig. 8:**
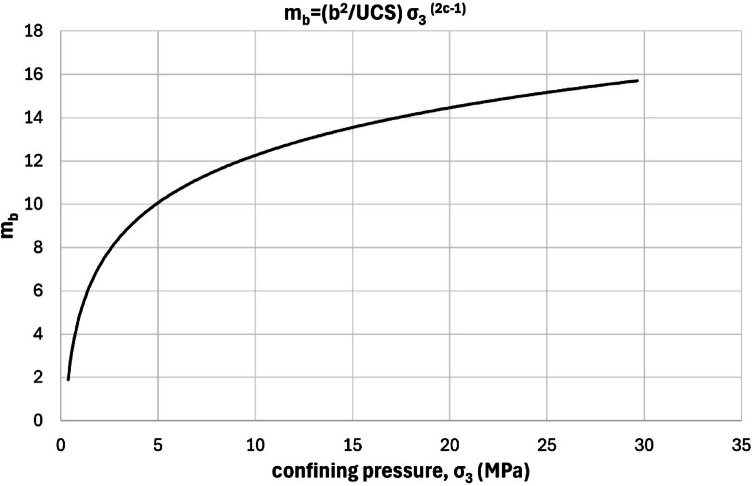
Relationship between residual m_b_ in function of confining pressure (σ_3_).

Figures 7 and 8 illustrates the dependency of *GSI* and m_b_ on confining stress, directly challenging the Walton et al.^18^ hypothesis, which suggests that *GSI* remains unaffected by confinement in intact rocks. The graph demonstrates a clear logarithmic trend, reinforcing the notion that *GSI* decreases at higher confining pressures. This insight underscores the nonlinear-value nature of rock mass strength under varying stress environments, making a compelling case for re-evaluating existing models that treat *GSI* as a constant-value. Such findings have profound implications for tunneling, slope stability, and underground excavation projects, where accurately predicting rock behavior under load is critical for design and safety assessments.

The x and z values provided in Table 3 further illustrate the variability in the calculated *GSI* across different rock samples. The average values of x = 10.96 and z = 25.78 reflect moderate rock strength, while the standard deviation suggests notable sample-to-sample variation. The *GSI* values span from 27 to 82 (average = 54), highlighting the diverse geological conditions represented in the dataset. This wide range suggests that certain samples possess high structural integrity (*GSI* above 70), while others display significant weakness (GSI below 40), emphasizing the need for site-specific analysis when applying these models in engineering practice.

**Table 3:** Independent material constants and measures *GSI* values related to the proposed approach in the function of the confining pressure.

No.	x	z	GSI
1	7.94	30.88	55
2	937	52.85	82
3	9.83	20.93	52
4	14.07	24.97	56
5	10.24	26.21	60
6	13.72	34.62	75
7	8.50	43.42	71
8	8.80	19.23	48
9	9.67	26.71	58
10	11.35	18.23	55
11	16.40	8.38	61
12	12.99	12.33	49
13	8.55	16.43	41
14	6.11	56.45	74
15	7.25	28.16	51
16	8.88	35.15	63
17	12.63	45.47	78
18	12.38	4.67	44
19	13.15	7.12	49
20	11.72	2.72	41
21	5.99	29.78	49
22	9.11	24.57	53
23	11.28	32.97	67
24	14.24	5.57	51
25	12.63	37.47	78
26	9.17	24.86	53
27	12.33	3.51	44
28	6.09	12.45	27
29	14.89	13.03	60
30	8.15	44.11	69
31	14.38	5.68	52
32	12.45	14.07	54
33	11.12	24.85	60
34	12.34	5.17	44
35	10.96	13.16	56
36	9.37	17.43	48
37	13.09	27.14	69
38	10.01	25.05	55
Av	10.82	23.05	57
STD	2.61	14.04	12
min	5.99	2.72	27
max	16.40	56.45	82

**Fig. 9:**
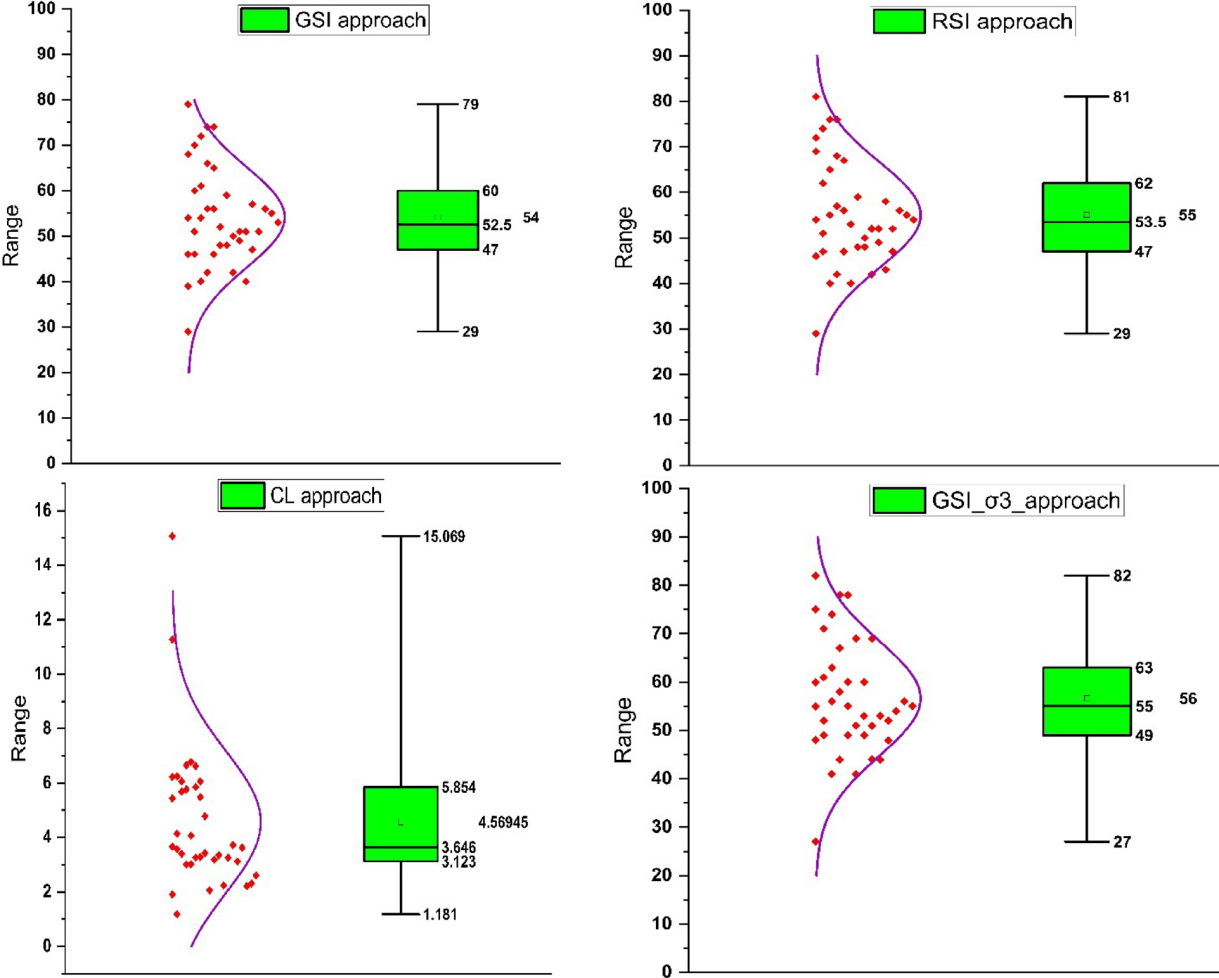
Box-and-whisker plots and scatter plots showing the distribution and correlation of key parameters used in residual strength modeling. Box plots display the median (center line), interquartile range (box limits), whiskers (1.5×IQR), and outliers (individual points outside whiskers).

Figure 9 presents box-and-whisker plots alongside scatter plots to illustrate the variability and distribution of key input and output parameters derived from the experimental analysis. The box-and-whisker plots summarize the statistical range, including the median, interquartile range, and outliers, for critical parameters such as *GSI*, *RSI*, and the λ parameter, which are used in residual strength modeling. These plots effectively highlight the moderate variability in the dataset, as reflected in the standard deviations reported in Tables 1, 2 and 3.

The accompanying scatter plots provide insight into the correlations between input variables (e.g., confining pressure, UCS) and output metrics like residual strength or model-specific constants (e.g., b and c). These scatter plots visually demonstrate trends, such as the non-linear relationship between confining pressure and residual strength, as well as the transition from brittle to ductile behavior under higher confining pressures. Such visualization reinforces the importance of confining pressure and geological parameters in influencing post-peak rock behavior. This figure is pivotal in linking the experimental results to theoretical models, emphasizing the heterogeneity of granitic rocks and the challenges of accurately predicting residual strength across varying geological conditions.

### Brittle-Ductile transition analysis

The relationship between deviator stress (σ_1_ − σ_3_) and confining pressure provides critical insights into the brittle-ductile transition of granitic rocks. This behavior is strongly influenced by material constants b and c, derived from regression models in Eq. (13), along with UCS and m_i_. These parameters reflect how the mechanical response evolves under varying confinement levels, transitioning from brittle failure to ductile deformation.

**Fig. 10:**
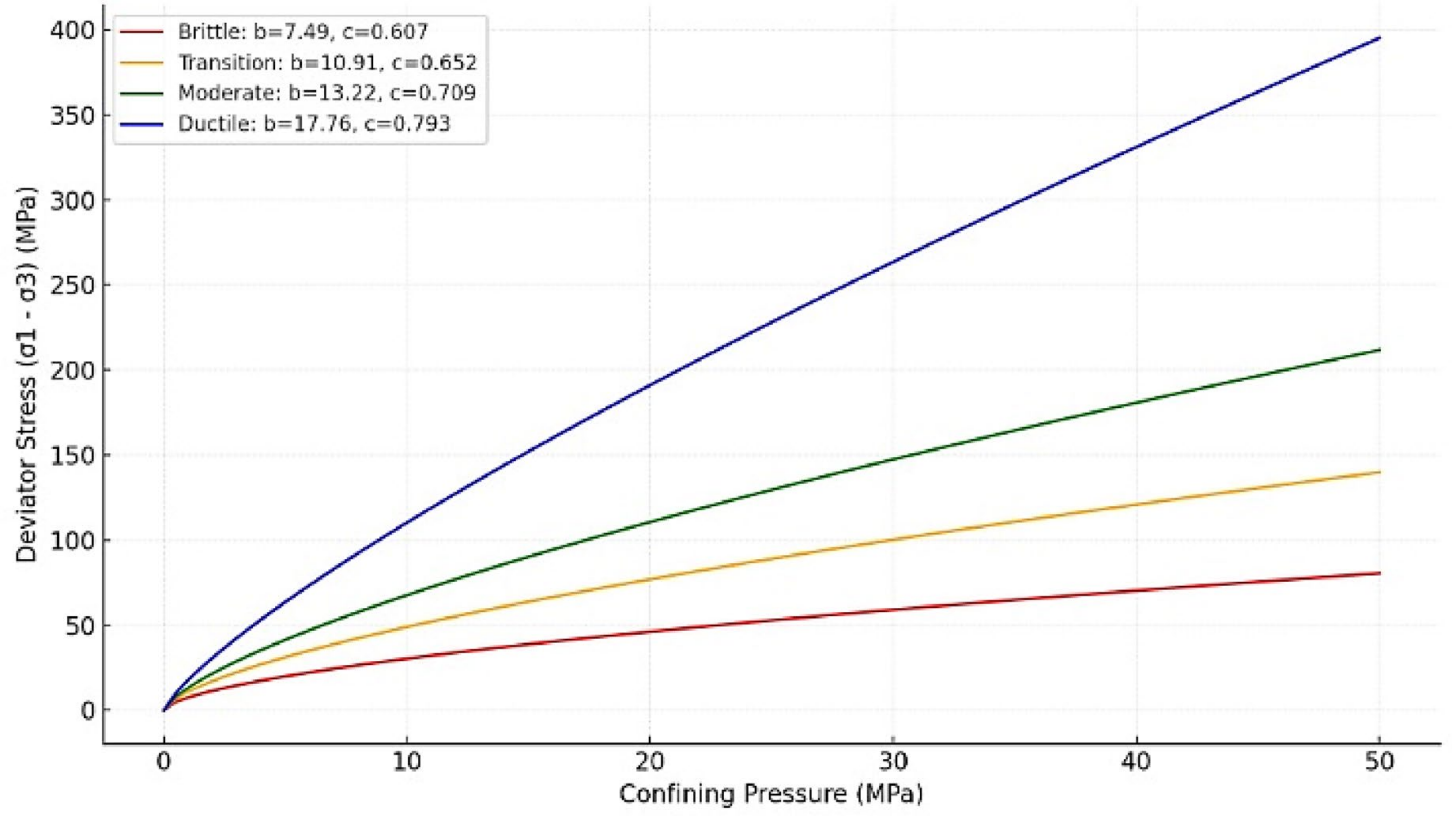
Brittle-ductile transition based on deviator stress (σ_1_ − σ_3_) and confining pressure (σ_3_).

According to Fig. 10, at low confining pressures, the response is dominated by brittle failure mechanisms. Samples characterized by low b and c values (e.g., b = 7.49, c = 0.607) exhibit steep stress gradients, indicative of rapid failure following peak strength. This behavior is consistent with rocks having lower UCS values. The red curve highlights brittle behavior with steep gradients and rapid stress drop post-failure. As confining pressure increases, the stress-strain response becomes more gradual, indicating a reduction in brittleness. Moderate b and c values (e.g., b = 10.91, c = 0.652) represent this transitional phase, where residual strength retains significant variability due to microcrack propagation and frictional effects. Intermediate curves (orange and green) capture the transition zone, bridging brittle and ductile behaviors. At higher confinement levels, samples with high b and c values (e.g., b = 17.76, c = 0.793) display ductile deformation, characterized by smooth and progressive stress increases. The ductile regime is observed predominantly in samples with higher UCS and m_i_, reflecting their capacity to sustain stress under confinement. The blue curve represents ductile behavior, where stress increases gradually, even at high confinement.

According to this figure, in brittle-dominant regimes, residual strength is lower because the rock loses cohesion rapidly after failure. In ductile regimes, residual strength is higher as frictional sliding mechanisms dominate over brittle fractures. Therefore, based on our analysis, dataset likely shows a trend where residual strength increases with confining pressure, which is consistent with σ_TR_-based transition predictions in the Davarpanah et al.^29^.

Moreover, Microcracking and cohesion loss are critical factors in the transition from brittle to ductile behavior. As confining pressure increases, microcracks propagate more slowly, and the rock undergoes plastic deformation rather than sudden brittle failure. This is consistent with findings from previous studies^30^, which show that cohesion loss is a key mechanism in the post-failure behavior of granitic rocks. The transition from brittle to ductile behavior can thus be understood as a shift from crack-dominated failure to frictional sliding and plastic flow. Therefore, according to the research of Vásárhelyi et al.^30^, a model to predict the confining pressure (σ_3_) at which the BDT occurs, based on the Geological Strength Index (GSI) and the material constant (m_i_) provided, Eq. 19:

19 $$\:{\sigma\:}_{3}^{*}=\frac{{\sigma\:}_{ci}(\frac{{\sigma\:}_{ci}}{10}-0.17)\sqrt{{(\frac{{\sigma\:}_{ci}}{10}-0.17)}^{2}+C}}{D}$$

Where:

- σ₃* represents the BDT stress.
- σc is the uniaxial compressive strength of the intact rock.
- m_i_ is a material constant related to the rock type.
- C and D are constants that vary based on the GSI value:
  - If GSI < 60, C = 46.24 and D = 23.12.
  - If GSI ≥ 60, C = 100 and D = 50.

By comparing empirical residual strength results with the σ_3_* values predicted by Eq. 19, we can assess the alignment between observed data and theoretical expectations. If residual strength measurements are significantly lower than the predicted σ₃*, it suggests that the rock mass is more susceptible to brittle failure under lower confining pressures. Conversely, if the residual strengths are higher or comparable to σ_3_*, the rock is likely to exhibit ductile behavior under similar conditions.

## Discussion

The residual strength of brittle rocks has been widely studied, with researchers using experimental, analytical, and numerical approaches to understand post-failure behavior under various confinement conditions. This study contributes to this body of research by analyzing the relationship between residual strength, confining pressure, and Geological Strength Index (*GSI*), Residual Strength Index (*RSI*), and the Cohesion-Loss (C-L) model. The results align with several key findings from previous studies while also challenging some conventional assumptions regarding rock mass behavior at residual states.

A major finding of this study is that residual strength increases with confining pressure, supporting previous studies on brittle-to-ductile transition mechanisms. Peng and Cai^3^ proposed that as confinement increases, cohesion loss is counterbalanced by frictional strengthening, leading to higher residual strength values. Similarly, Gao and Kang^5^ demonstrated that residual strength increases more sharply than peak strength under elevated confining pressures in coal-bearing formations, a trend also observed in this study for granitic rock.

Additionally, He et al.^2,28^ applied linear elastic fracture mechanics to predict the brittle-ductile transition (BDT) point, concluding that microcrack interactions and slip friction mechanisms govern residual strength evolution. The findings of the present study support this argument, as the non-linear regression model (₁ – ₃ = b₃^c^) confirms that deviator stress follows an increasing but diminishing trend as confinement rises. This aligns with Mahmutoglu and Şans^25^, who reported that residual cohesion increases significantly under high confinement, while the internal friction angle decreases.

In this study, the Geological Strength Index (*GSI*) and Residual Strength Index (*RSI*) models yielded comparable estimates of rock mass quality (mean values: *GSI* = 54, *RSI* = 55), consistent with the findings of Walton et al.^17,18^. However, Cai et al.^1^ originally proposed that *GSI* remains largely independent of confining pressure, assuming that rock mass characterization is constant-value. The present study challenges this assumption by demonstrating that *GSI* exhibits a logarithmic dependency on confining pressure. This is further supported by Davarpanah et al.^29^, who showed that brittle-to-ductile transitions alter *GSI* values over time as deformation progresses.

Although the GSIr concept by Cai et al.^1^ provides a useful framework for linking residual strength to the Hoek–Brown criterion, its application as a material parameter should be treated with caution. GSI was developed to classify rock mass structure and surface conditions, and using it to represent post-failure degradation may oversimplify complex geological processes, particularly in pre-sheared or tectonically damaged zones. In such cases, the GSI value itself may already reflect a residual condition. Therefore, while GSIr can be used as a modeling tool, its physical interpretation must be considered carefully within geological context.

The RSI model, introduced by Walton et al.^17,18^, simplifies the Hoek-Brown residual failure criterion by setting the constant “s” to zero, making it more suitable for post-failure analysis. The RSI approach provides a good fit for residual strength data across various rock types, reinforcing its applicability in residual strength estimation^36^. The present study confirms the effectiveness of RSI but also highlights that RSI values tend to increase at higher confinement, suggesting that the model should be adapted to account for confinement-dependent behavior in fractured rock masses.

The Cohesion-Loss (C-L) model proposed by Peng and Cai^3^ introduces a dimensionless parameter (λ) to quantify cohesion loss, providing an alternative to traditional failure criteria. The present study found that λ values varied significantly (1.181 to 15.069), indicating high variability in post-failure behavior, which aligns with Tiwari et al.^15^, who noted that residual strength variability is largely controlled by mineralogical composition and pre-existing fracture networks.

The implications of these findings extend beyond model calibration. They offer a mechanistic interpretation of the brittle–ductile transition in granitic rocks. Samples with lower b and c parameters exhibited rapid stress drops after failure, indicative of brittle behavior, while those with higher values maintained more gradual stress–strain curves, consistent with ductile deformation. This transition is not merely a function of stress state but is mediated by the progressive mobilization of frictional strength and the degradation of cohesive bonds. The transition thresholds observed in this study closely match those predicted by Davarpanah et al.^29^, who provided a theoretical framework for identifying σ₃*, the confining pressure at which the transition occurs. Furthermore, the relationship between increasing λ values and ductile response confirms the central role of cohesion loss in dictating deformation behavior, as previously proposed by He et al.^28^.

This study also aligns with Hou and Cai^19^, who explored post-peak stress-strain behavior of brittle rocks and found that residual strength models must account for localized fracture mechanics. The Hoek-Brown residual strength criterion, while widely used, does not explicitly model the progressive loss of cohesion, leading researchers to propose alternatives such as the Joseph-Barron (J-B) model^23^, which introduces a stiffness parameter for post-failure behavior.

Furthermore, Lin et al.^16^ developed a strain-softening model based on the Hoek-Brown criterion, which captures the progressive reduction in rock strength due to microcracking. The present study’s findings align with this approach, confirming that traditional peak strength models must be modified to include strain-softening effects in brittle rocks.

Microstructural studies have demonstrated that residual strength is closely tied to fracture density and mineral deformation. Zhao et al.^4^ investigated how microcracks influence compressive strength and found that residual strength is highly dependent on pre-existing flaws and their orientation relative to loading direction. The results of the present study suggest that mineralogical heterogeneity contributes to the variability in residual strength parameters, further reinforcing these observations.

Additionally, Ivars et al.^11^ developed the Synthetic Rock Mass (SRM) model, which uses discrete element simulations to assess how jointed rock masses behave under stress. Their findings suggest that residual strength cannot be predicted solely based on intact rock properties, as joint spacing and gouge content play significant roles in determining shear strength post-failure. This observation is consistent with the variability in GSI and λ values observed in the present study, indicating that future work should incorporate discrete fracture models to better capture rock mass behavior in situ.

The Hoek-Brown damage factor (D factor) is typically used to account for the influence of weathering, excavation disturbance, and structural weaknesses on rock mass strength, particularly in empirical rock mass classifications. However, in this study, we employed a modified form of the Hoek-Brown criterion tailored to our specific research objective—examining the effect of confining pressure (σ₃) on residual strength. Through nonlinear regression analysis, a relationship between deviator stress and confining pressure was established, enabling the determination of residual Geological Strength Index (GSI) as a function of σ₃. The findings indicate that residual GSI exhibits a logarithmic increase with rising confining pressure, demonstrating that it is not a fixed material constant but rather a pressure-dependent parameter. This observation suggests that the residual strength behavior is more accurately represented through this nonlinear approach rather than by incorporating an empirical disturbance factor such as D. Since the impact of confining pressure on residual strength is inherently captured within our modified Hoek-Brown model, an explicit consideration of the D factor is unnecessary in this context. By focusing on the direct σ₃-residual strength relationship, this study provides a more precise assessment of rock mass behavior without relying on additional empirical adjustments related to disturbance effects.

## Limitations of the study

While the study presents a strong framework for understanding residual strength evolution in granitic rocks, several limitations should be noted:

- The applied models, including the *GSI*, *RSI*, and cohesion-loss approaches, are grounded in empirical relationships, relying on statistical fitting rather than direct physical observations of fracture progression. Also, the Hoek–Brown failure criteria-based *GSI* and *RSI* approaches assume that rock mass properties remain uniform across scales, which may not hold true for highly anisotropic formations or heavily fractured rock masses.
- The triaxial tests were conducted under controlled laboratory conditions, which do not fully capture the effects of in situ stress redistribution, pore pressures, and long-term creep behavior. Natural rock masses typically include pre-existing discontinuities, joint networks, and weathered zones, which were not explicitly represented in the tested samples. This might restrict the direct applicability of the findings to large-scale engineering contexts.
- The study does not explicitly analyze microcrack evolution or mineralogical changes due to increasing confining pressure. Techniques such as Scanning Electron Microscopy (SEM) or X-ray Diffraction (XRD) could provide additional insights into progressive crack closure and mineral deformation at the microscale.

## Conclusions

This study examined the residual strength behavior of granitic rocks from Bátaapáti, Hungary, with a focus on the interplay between the Geological Strength Index (*GSI*), Residual Strength Index (RSI), and confining pressure. Through extensive multiple failure state (MFS) triaxial testing, the research provided critical insights into post-failure rock behavior, revealing that residual strength is strongly influenced by confining pressure, transitioning from brittle failure to ductile deformation as confinement increases. The results underscore the importance of considering confining pressure variations in residual strength assessments rather than assuming constant material properties.

Three previously presented key approaches plus a new proposed model evaluated for predicting residual strength: the *GSI*-based approach, the *RSI* method, and the cohesion-loss (C-L). The GSI and RSI approaches provided comparable estimates of rock mass quality, with mean values of 54 and 55, respectively, and moderate variability. The cohesion-loss model, which quantifies the degradation of cohesion post-failure through the parameter λ, displayed a broader range of values (1.181 to 15.069), suggesting significant heterogeneity in the post-failure behavior of the granitic rock samples. These findings highlight the necessity of selecting appropriate models that account for material-specific variability when predicting residual strength.

Nonlinear regression analysis of deviator stress versus confining pressure yielded key material constants (b and c), with average values of 11.38 and 0.693, respectively.

The findings of this study on the residual strength of granitic rocks have direct implications for various engineering applications, particularly in tunneling, mining, and slope stability. Understanding the interplay between Geological Strength Index (*GSI*), Residual Strength Index (*RSI*), and confining pressure provides a framework for improving design and safety measures in rock engineering.

Furthermore, the study introduced a modified framework to predict the brittle–ductile transition threshold using Hoek–Brown parameters and GSI-dependent empirical constants. The strong agreement between the theoretical transition stress (σ₃*) and observed mechanical behavior across samples validates this approach and highlights its potential for broader application in geotechnical assessments.

The study’s results indicate that residual strength increases with confining pressure, suggesting that tunnels at greater depths, where natural confinement is higher, may have more stable excavation conditions. Engineers can use these findings to optimize rock support systems by tailoring reinforcement methods based on depth and expected stress conditions. Also, the proposed models can help predict the extent of post-failure deformation around tunnels. This is crucial for designing tunnel linings and support structures to prevent excessive deformations and collapses in fractured rock masses.

In deep mining operations, rock bursts and pillar failure are significant risks. The findings provide a more reliable way to predict residual strength, which is essential for designing stable mine pillars and stopes, reducing the likelihood of catastrophic failures. Moreover, the study’s proposed regression models and strength prediction approaches can be incorporated into numerical simulations for mine planning. This allows for more accurate risk assessments of excavation-induced stress redistribution and long-term mine stability. The correlation between residual strength and confining pressure provides insights into fracture mechanics, helping to anticipate zones prone to shear failure, which is critical in both open-pit and underground mining.

The study confirms that fractured and weathered granitic rock masses have significantly lower residual strengths, especially under low confinement conditions. Engineers can use this information to refine slope stability models and ensure more precise assessments of landslide risks in hilly or mountainous terrains. In cases where slopes have already failed, the study’s insights into post-failure strength help in designing remedial measures by accurately estimating the residual load-bearing capacity of the failed rock mass.

## Data Availability

The datasets used and/or analyzed during the current study available from the corresponding author on reasonable request.
